# Reviewed article: Nogueira MRN, Braga HFGM, Sousa VTS, Maciel NS, Melo ESJ, Vasconcelos PF, Sousa LB. HU-UFGD/Ebserh, Setor de gerenciamento da pesquisa e inovação tecnológica. Epidemiol Serv Saude. 2025:34;e20240493

**DOI:** 10.1590/S2237-96222025v34e20240493.b

**Published:** 2025-06-20

**Authors:** Dayse Paiao

**Affiliations:** Prefeitura Municipal de Porto Murtinho, Secretaria Municipal de Saúde

**Table:** 

Rating	Excellent	Good	Average	Below average	Poor
Interest		✓			
Quality			✓		
Originality			✓		
Overall			✓		

**Table:** 

Questionnaire	Yes	No	Not applicable
Does the manuscript contain new and significant information to justify publication?	✓		
Does the Abstract (Summary) clearly and accurately describe the content of the article?		✓	
Is the problem significant and concisely stated?		✓	
Are the methods described comprehensively?		✓	
Are the interpretations and conclusions justified by the results?		✓	
Is adequate reference made to other work in the field?	✓		

## Manuscript Structure

Length of article is: Adequate

Number of tables is: Too little

Number of figures is: Too little

Please state any conflict(s) of interest that you have in relation to the review of this paper (state “none” if this is not applicable): None

## Comments

A pesquisa contribui para o SUS ao identificar a prevalência do Vírus da Imunodeficiência Humana (HIV) na população privada de liberdade, no Brasil e em suas regiões, o que pode auxiliar na elaboração de políticas públicas de saúde direcionadas à prevenção e ao tratamento da doença de forma regionalizada.

Entretanto, alguns pontos causaram preocupações com o artigo, estas são:

1) O resumo precisa ser revisado pois os resultados apresentados devem estar apoiados por dados numéricos.

2) A introdução não embasa a temática de forma robusta, deixando insipiente a importância de conhecer a prevalência do HIV em privados de liberdade, o autor não descreve a prevalência no mundo, no Brasil e entre suas regiões para posteriores comparações;

3) Na metodologia não foi descrito a caracterização da população, o que ajudaria a entender o cenário carcerário brasileiro.

4) Nos resultados não é apresentado as prevalências a nível nacional, regional e por gênero, este dado é importante para fazer comparações entre as regiões.

5) A discussão deve ser revista, os motivos associados ao aumento da prevalência de HIV no Brasil não ficaram claras, sobretudo a alta prevalência entre as mulheres, segundo a literatura, quais as hipóteses justificam as altas prevalências e a tendência de aumento.

